# Tall fescue cultivar and fungal endophyte combinations influence plant growth and root exudate composition

**DOI:** 10.3389/fpls.2015.00183

**Published:** 2015-04-09

**Authors:** Jingqi Guo, Rebecca L. McCulley, David H. McNear

**Affiliations:** ^1^Rhizosphere Science Laboratory, Department of Plant and Soil Sciences, University of KentuckyLexington, KY, USA; ^2^Grassland Ecology Laboratory, Department of Plant and Soil Sciences, University of KentuckyLexington, KY, USA

**Keywords:** *Epichloë coenophiala*, fungal endophyte, root exudates, plant growth, tall fescue

## Abstract

Tall fescue [*Lolium arundinaceum* (Schreb.)] is a cool-season perennial grass used in pastures throughout the Southeastern United States. The grass can harbor a shoot-specific fungal endophyte (*Epichloë coenophiala*) thought to provide the plant with enhanced resistance to biotic and abiotic stresses. Because alkaloids produced by the common variety of the endophyte cause severe animal health issues, focus has been on replacing the common-toxic strain with novel varieties that do not produce the mammal-toxic alkaloids but maintain abiotic and biotic stress tolerance benefits. Little attention has been given to the influence of the plant-fungal symbiosis on rhizosphere processes. Therefore, our objective was to study the influence of this relationship on plant biomass production and root exudate composition in tall fescue cultivars PDF and 97TF1, which were either not infected with the endophyte (E-), infected with the common toxic endophyte (CTE+) strain or with one of two novel endophytes (AR542E+, AR584E+). Plants were grown sterile for 3 weeks after which plant biomass, total organic carbon, total phenolic content and detailed chemical composition of root exudates were determined. Plant biomass production and exudate phenolic and organic carbon content were influenced by endophyte status, tall fescue cultivar, and their interaction. GC-TOF MS identified 132 compounds, including lipids, carbohydrates and carboxylic acids. Cluster analysis showed that the interaction between endophyte and cultivar resulted in unique exudate profiles. This is the first detailed study to assess how endophyte infection, notably with novel endophytes, and tall fescue cultivar interact to influence root exudate composition. Our results illustrate that tall fescue cultivar and endophyte status can influence plant growth and root exudate composition, which may help explain the observed influence of this symbiosis on rhizosphere biogeochemical processes.

## Introduction

Tall fescue [*Lolium arundinaceum* (Schreb.) Darbysh. = *Schedonorus arundinaceus* (Schreb.) Dumort., formerly *Festuca arundinacea* Schreb. var. *arundinacea* Schreb.] is a perennial, cool season bunchgrass native to Eurasia and North Africa (Gibson and Newman, 2001), but now also occurs in North America, Australia, and New Zealand (Young et al., 2013). The majority of commercial tall fescue cultivars are allohexaploid (2n = 6x = 42) (Humphreys et al., 1995). Significant heterozygosity and high self-incompatibility result in tall fescue cultivars being populations of genetically unique individuals (Xu et al., 1994). “Kentucky-31” is one of the most common tall fescue varieties in the North America, and can host a shoot specific fungal endophyte (*Epichloë coenophiala* formerly *Neotyphodium coenophialum*). *E. coenophiala* is an asexual species (heteroploid) transmitted vertically by infecting plant seeds (Clay and Schardl, 2002). The hyphae of *E. coenophiala* grow into developing ovules and seeds on symbiotic tall fescue plants and then after seed germination colonize the plant leaf sheaths and stems via intercalary growth, but are sparse or not present in the roots (Clay, 1988; Tan and Zou, 2001; Timper et al., 2005; Christensen et al., 2008; Schardl, 2010). *E. coenophiala* is an obligate symbiotic fungus, surviving by absorbing water, amino acids and sugars produced by the plant (Malinowski and Belesky, 2000; Sabzalian and Mirlohi, 2010). In return, fungal-produced primary or secondary metabolites serve to protect the plant from herbivores and enhance tolerance to climactic and edaphic stresses, ultimately improving overall plant fitness (Bouton et al., 1993; Malinowski and Belesky, 2000).

Secondary metabolites, such as alkaloids and phenolic compounds, are ecologically important components produced by the endophyte and the host tall fescue plant, respectively. *Epichloë* species produce four main classes of alkaloids including ergot alkaloids, indole diterpenes, 1-aminopyrrolizidines and peramine (Bush et al., 1993, 1997; Schardl, 2009). N-formaylloline, ergovaline and peramine are produced in most *E. coenophiala* infected tall fescue (Schardl, 2009). The alkaloids (specifically the ergot alkaloids) are responsible for an estimated $600 million in annual beef cattle losses due to animal weight loss and reduced calf births (Morgan et al., 2005)—an ailment commonly referred to as “fescue toxicosis.” Fescue toxicosis has also been linked to animal health issues in horses (Putnam et al., 1991; Christiansen et al., 2007), sheep (Burke et al., 2002), goats (Smith et al., 2004), and Canadian geese (Conover and Messmer, 1996).

Because of the impact on animal production, efforts have been made to replace common toxic strains of the endophyte (CTE+ – toxic to mammals and insects) with so-called “novel” non-mammal-toxic strains. For example, Novel endophyte AR542 and AR584 have been inserted into tall fescue cultivar “Jesup” and “Texoma” and commercialized as “Jesup” MaxQ and “Texoma” MaxQ II, respectively Bouton et al., 2002; Hopkins et al., 2011. Novel endophyte strains have been shown to provide the advantages conferred by CTE+ strains to the plant (Bouton et al., 1993), but they don't produce the ergot alkaloids responsible for fescue toxicosis (Parish et al., 2003; Phillips and Aiken, 2009). In addition, forage yield of “Texoma” MaxQII has been shown to be 20% higher than “Jesup” MaxQ (Hopkins et al., 2011). How the CTE+ strains of the fungus interact with tall fescue to alter belowground processes has received some attention (Malinowski and Belesky, 1999a,b, 2000; Malinowski et al., 2000; Franzluebbers and Stuedemann, 2005; Iqbal et al., 2012; McNear and McCulley, 2012); however, less is known about how the interaction of novel fungal genotypes and tall fescue cultivars influence rhizosphere biogeochemical processes such as nutrient cycling, microbial community structure and function, and root exudate production.

While it is clear that the alkaloids produced by *E. coenophiala* have biological activity, it is unlikely that they are solely responsible for the wide range of below-ground biogeochemical effects associated with endophyte infection, such as the observed increases in soil C and N storage in highly endophyte infected tall fescue pastures of the southeastern U.S. (Franzluebbers et al., 1999a; Franzluebbers, 2006; Iqbal et al., 2012). The alkaloids are found only within the plant and have not been identified in exudates released from plant roots into the rhizosphere. There is only one study (Franzluebbers and Hill, 2005) where alkaloids were found in surface soils in pastures dominated by CTE+ tall fescue, but this may have been due to alkaloids leaching from decomposing leaf litter (Assuero et al., 2006), in which they can persist in measurable concentrations for up to 50 days (Siegrist et al., 2010). There are a few studies that have found differences in root exudate composition resulting from infection of tall fescue with the CTE+ strain. Van Hecke et al. (2005) found more carbohydrates and organic carbon in root exudates of CTE+ than endophyte-free (E-) tall fescue (Van Hecke et al., 2005). Malinowski et al. (1998); Malinowski and Belesky (1999a) also found that exudates from CTE+ tall fescue contained higher amounts of phenolic compounds compared to E- tall fescue which they attributed to increased P acquisition by CTE+ plants (Malinowski et al., 1998; Malinowski and Belesky, 1999a). The effect of tall fescue genotype was also shown to be a significant factor influencing root exudate composition based on the indirect observation that exudates from Grassland Flecha (a Mediterranean tall fescue) had greater Cu^2+^—binding activity compared to a continental type tall fescue—Jesup—regardless of endophyte status which the authors attributed to cultivar differences in total phenolic compound exudation (Malinowski et al., 2004).

Changes in root exudate amount and composition resulting from endophyte infection may contribute directly to endophyte associated differences in C and N quantities in pasture soils by altering inputs to these pools, or indirectly by altering the structure and function of the soil microbial community which controls the processing and turnover of these soil pools. Root exudates are comprised of carbon compounds such as organic, fatty, and amino acids, sugars, phenolics, and proteins (Jones et al., 2004; Schmidtke, 2005; Broeckling et al., 2008). Many of these compounds have been positively correlated with microbial activity (Vale et al., 2005). There are numerous examples in the literature of other cool season grasses in which fungal endophyte infection has resulted in measurable shifts in microbial community structure and function (Chu-Chou et al., 1992; Van Hecke et al., 2005; Jenkins et al., 2006; Casas et al., 2011). However, variability in aboveground fungal endophyte effects on belowground parameters have also been observed, with some sites having stronger effects than others (Iqbal et al., 2012).

Although there is some evidence to suggest that shoot-specific fungal endophytes in tall fescue play a role in altering classes of chemical compounds in root exudates (e.g., phenolics, carbohydrates, etc.), there are currently no detailed studies on whether this symbiosis influences the release of specific chemical compounds. Further, all of the work to date on the chemical composition of root exudates from tall fescue has focused on CTE+ tall fescue, and none have examined the influence of the newer novel endophyte strains or their interaction with different tall fescue cultivars. Our objective was therefore to evaluate how fungal endophyte strain interacts with fescue cultivar to affect plant growth and root exudate composition. We hypothesized that both endophyte status and tall fescue cultivar would have significant influences on plant biomass production and root exudate composition. Because alkaloids have never been identified in root exudates, our focus was on identifying other exogenous compounds more likely responsible for influencing key biogeochemical processes such as nutrient cycling, microbial community structure and function.

## Materials and methods

### Seed preparation and pure culture growth

Endophyte-free (E-), common toxic endophyte (CTE+), and novel endophyte (AR542E+ and AR584E+) infected tall fescue cultivars PDF and 97TF1 were obtained from the Samuel Roberts Noble Foundation (Ardmore, OK). Both cultivars were selected from tall fescue collections originating from the Ardmore, OK area and accessions from the USDA Plant Introduction system. The Noble Foundation created the endophyte statuses using standard techniques for both cultivars (Hopkins et al., 2011). The two fescue cultivars were chosen because both contained the full complement of endophyte statuses, sufficient seed was available for all combinations, and they were part of a 6 year grazing study at the University of Kentucky Spindletop Research Farm. Both cultivars were also being evaluated for release to the seed market and were therefore likely to be grown in the USA soon (e.g., PDF-AR584E+ is currently available to forage producers as Texoma MaxQ II™). Finally, novel endophytes AR542 and AR584 were chosen because they are the most common novel endophyte strains in tall fescue in the United States and abroad at present, though acreage of both is still significantly less than CTE+ tall fescue (Young et al., 2013). They also produce significantly different alkaloid profiles than CTE+ (Takach and Young, 2014). No analysis was done to confirm that the endophyte was isolated to only the shoots. However, there is ample evidence in the literature to show that *E. coenophiala* is a shoot specific fungal endophyte (Clay, 1988; Tan and Zou, 2001; Timper et al., 2005; Christensen et al., 2008; Schardl, 2010).

For this study, plants were grown hydroponically under pure culture conditions to collect root exudates for characterization. All materials were autoclaved prior to use and kept sterile until needed. Seeds were sterilized by counting and weighing 125 seeds per replicate into 50 ml falcon tubes (Barnstead International, Dubuque, Iowa), adding 40 mL of a 10% bleach and Tween-20 mixture and then placing them on an orbital shaker set at 60 rpm for 30 min. After sterilization, the seeds were rinsed three times with ultrapure double deionized (DDI) water (Barnstead International, Dibuque, Iowa) and then transferred, under sterile conditions, to a floating polypropylene explant holder inside a Growtek™ chamber (Krackeler Scientific, Inc, Albany, NY) containing 80 mL of filter-sterilized liquid minimal nutrient media (MNM). The growth vessels were then placed randomly on a stagnant rotary shaker (Barnstead International, Dubuque, Iowa) under cool white fluorescent light (100 mmol m^−2^s^−1^; 16 h light/ 8 h dark) until seedling germination. After about 1 week, when the seeds had germinated and were adequately anchored, the shaker was turned on and maintained at 65 rpm for the entirety of the experiment. A period without shaking was necessary at the start of the experiment to prevent seed “pile up” and ensure an even distribution of seeds across the explant holders. The bottom of the vessels was wrapped with aluminum foil to minimize light penetration in the rooting zone and the possible photodegradation of exudate components. Experiments were set up in a randomized complete block design with 3 replicates per treatment [endophyte (4) × cultivar (2)] for a total of 24 culture vessels per experiment. Experiments were repeated 5 times resulting in 15 replicates (~1875 plants) per treatment.

### Exudate collection and plant biomass determination

After 21 days the nutrient solutions were collected and passed through 0.45 μm nylon filter using a Stericup® filter unit (Millipore Corporation, Billerica, MA) to remove any large root debris or sloughed cells. The solution was then divided into four parts: (1) 1 ml for phenolic assay; (2) 2 ml for pH analysis; (3) 3 ml for TOC analysis (27 ml sterilized water was added); (4) remainder was saved in 50 ml centrifuge tubes and stored with desiccant at −20°C for analysis via GC-TOF MS. The grasses were then separated into root and shoot, counted and weighed after drying in the oven for 2 days at 65°C. Plant biomass used in this paper was calculated on a per 1000 plant basis as follows: plant biomass = (biomass of each vessel/tiller number)^*^1000.

### Exudate total organic carbon and phenolic content

Total organic carbon (TOC) within the raw nutrient solutions was determined by heating the samples to 680°C using the platinum catalyst in a Shimadzu TOC-V combustion analyzer (Shimadzu Corporation, Kyoto, Japan) equipped with an infrared gas analyzer (NDIR) for carbon dioxide detection. The system is equipped with an auto-dilution system which was used to create a five point standard calibration curve before and after each run. Total phenolic content of exudates were determined following the procedure described in Arnow (1937). Briefly, 0.5 ml of root exudate was mixed with 91 μl derivitizing reagents and analyzed at 500 nm on a Thermo Spectronic Genesys 20 spectrophotometer (Thermo Electron Scientific Instruments Corp., Madison, WI, USA). The derivitizing reagent was comprised of 0.5 M HCL, 1.0 M sodium hydroxide, 1.45 M sodium nitrite and 0.41 M sodium molybdate. Quantification of total phenolics was achieved using a calibration curve generated using different concentrations of gallic acid. TOC and phenolic content were reported on a per gram root mass basis.

### Exudate composition via gas chromatography time of flight-mass spectrometry (GC-TOF MS)

In preparation for GC analysis, root exudates were extracted in acetonitrile, dried down in a speedvac and then derivitized for GC TOF-MS (Sana et al., 2010). An Agilent 6890 gas chromatograph coupled to a Pegasus IV time-of-flight mass spectrometer (Agilent, Böblingen, Germany) was used to analyze the root exudate composition. A Gerstel CIS4 with dual MPS injector with a multipurpose sample (MPS2) dual rail was used to inject 0.5 μL of the sample into the Gerstel CIS cold injection system (Gerstel, Muehlheim, Germany). The injector was operated in splitless mode with a flow rate of 10 μl/s, opening the split vent after 25 s and then increasing the temperature from 50°C to 250°C at a rate of 12°C/s. For separation, a 30 m long, 0.25 mm i.d. Rtx-5Sil MS column was used with an additional 10 m integrated guard column (0.25 μm of 5% diphenyl film and an additional 10-m integrated guard column; Restek, Bellefonte, PA). The carrier gas was 99.9999% pure Helium with built-in purifier (Airgas, Radnor PA) set at constant flow rate of 1 ml/min. The oven temperature was held constant at 50°C for 1 min and then ramped at 20°C/min to 330°C at which it was held constant for 5 min. Mass spectrometry was performed on a Pegasus IV TOF mass spectrometer (St. Joseph, MI) with the transfer line temperature between gas chromatograph and mass spectrometer maintained at 280°C, electron impact ionization energy of −70 eV and an ion source temperature of 250°C. MS data were acquired from m/z 85–500 at 17 spectra s^−1^ controlled by the LecoChromaTOF software vs. 2.32 (St. Joseph, MI). Data were preprocessed immediately after acquisition, stored as.cdf files and then automated metabolite annotation was performed using the BinBase metabolic annotation database (Fiehn et al., 2005). The relative abundance of the compounds was calculated via peak height normalized to the sum intensity of all identified peaks.

### Statistics

Biomass, phenolics and TOC data were log transformed if not normally distributed prior to analysis. Proc GLM statistical tests were run in SAS 9.3 (SAS Institute Inc, Cary, NC, USA) to assess the significance (*P* < 0.05) of the fixed main effects of endophyte status (E-, CTE+, AR542E+, AR584E+), fescue cultivar (PDF, 97TF1) and their interaction. For parameters with significant main effects or interactions, Student's *t*-test was used to conduct mean comparisons. All root exudate composition data were log transformed before subjecting to multivariate analyses. ANOVAs were run on individual compound relative abundances and grouped root exudate compounds in JMP 10.0.0 (SAS Institute Inc., Cary, NC, USA). Hierarchical clustering was used to group the identified root exudate compounds into clusters using a Ward's minimum variance method (Ward, 1963). The results are presented as dendrograms, and color maps were generated after clustering to show how the metabolite levels vary by endophyte status and cultivar.

## Results

### Plant biomass

**Figure 1:**
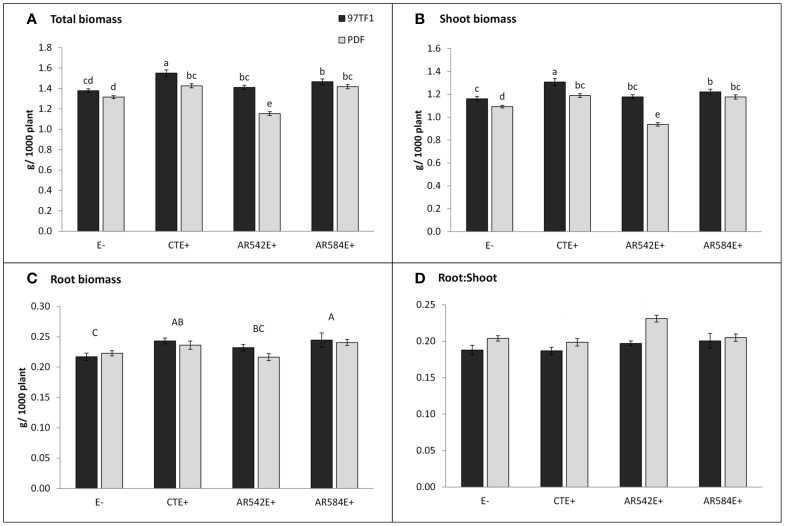
**Influence of endophyte status and tall fescue cultivar (PDF and 97TF1) on plant biomass. (A)** Total biomass, **(B)** shoot biomass, **(C)** root biomass, and **(D)** root: shoot. E- represents endophyte free tall fescue; CTE+ represents common toxic endophyte infected tall fescue; AR542E+ and AR584E+ represent novel endophyte infected tall fescue. Different lower case letters indicate significant differences (Student's *t*-test, *P* ≤ 0.05) among the combinations of cultivar and endophyte status within a panel. Different capital letters indicate significant differences (Student's *t*-test, *P* ≤ 0.05) across endophyte statuses when the cultivar x endophyte interaction was not significant. For root: shoot **(D)**, there was no effect of endophyte status or interaction with cultivar, but the two cultivars differed from each other (PDF > 97TF1).

Cultivar 97TF1 tended to produce more total plant biomass than PDF, particularly when both cultivars were infected with CTE and AR542 (Table 1, Figure 1A). In PDF, there were significant differences between the novel endophyte infected individuals, with AR542E+ producing less total plant biomass than AR584E+ (Figure 1A). For 97TF1, CTE+ produced more total biomass than the other endophyte statuses. Shoot mass was significantly affected by tall fescue cultivar, endophyte status and their interaction (Table 1). Cultivar 97TF1 had 6–17% more shoot mass than PDF, depending on the endophyte status, with the exception of novel endophyte AR584E+ infected individuals, where cultivars were similar (Figure 1B). For both 97TF1 and PDF, CTE+, and AR584E+ combinations resulted in a significant increase (+5–12%) in shoot mass relative to E-. For PDF, AR542E+ individuals had 15% less shoot mass than E-, and for 97TF1, AR542E+ individuals were intermediate to E- and AR584E+ (Figure 1B, Table 1).

**Table 1:** **Univariate ANOVA testing tall fescue cultivar, endophyte status and their interaction on fescue biomass parameters**.

**Effect**	**Df_n,d_**	**Root mass**		**Shoot mass**		**Total mass**		**Root:shoot**	
		**F**	**P**	**F**	**P**	**F**	**P**	**F**	**P**
Cultivar	1,1	1.68	0.21	47.45	**<0.0001**	47.2	**<0.0001**	11.98	**0.002**
Endophyte	3,3	5.96	**0.003**	24.03	**<0.0001**	26.8	**<0.0001**	3.97	0.09
Cultivar × Endophyte	3,3	0.86	0.48	6.46	**<0.0002**	6.8	**<0.0001**	1.97	0.14

*Significant effects (P ≤ 0.05) are shown in bold*.

Endophyte status, but not cultivar or their interaction, had a significant influence on average root mass (Table 1; Figure 1C). There was a significant difference in root mass production between the novel endophyte individuals: AR584E+ produced significantly more root biomass than AR542E+ infected individuals (Figure 1C). Notably, as with shoot biomass, the PDF/AR542E+ individuals produced the least root biomass, on par with E- material (Figure 1C).

Differences in root and shoot biomass reflected in the ratio of root mass to shoot mass were significantly influenced by tall fescue cultivar only (Table 1; Figure 1D). Overall, cultivar PDF had significantly higher root to shoot ratios than 97TF1 (0.21 vs. 0.19, respectively, averaged across endophyte status).

### Carbon and total phenolic content in root exudates

**Figure 2:**
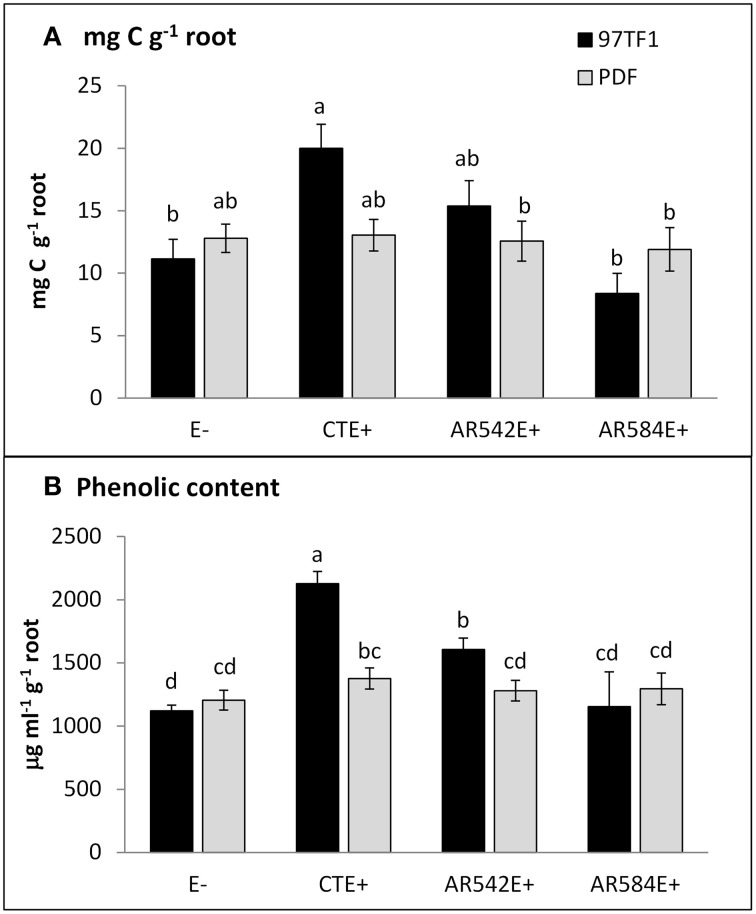
**Influence of endophyte status and tall fescue cultivar on carbon and phenolic content. (A)** mg C g^−1^ root, **(B)** phenolic content g^−1^ root. 97TF1 and PDF represent tall fescue cultivars. E- represents endophyte free tall fescue; CTE+ represents common toxic endophyte infected tall fescue; AR542E+ and AR584E+ represent novel endophyte infected tall fescue. Different letters indicate significant differences (Student's *t*-test; *P* ≤ 0.05) across endophyte statuses and tall fescue cultivar combinations.

CTE+ infected grasses tended to exude more total carbon than other individuals, largely due to the amounts released from 97TF1/CTE+ individuals (Table 2 and Figure 2A). In 97TF1, the CTE+ individuals had nearly twice the mg C g^−1^ root than E- or novel endophyte infected individuals (Figure 2A), while AR584E+ released significantly less C than the AR542E+ individuals (Figure 2A). Infection with CTE also stimulated phenolic release in 97TF1 compared to PDF and compared to the other cultivar/endophyte statuses (Figure 2B).

**Table 2:** **Univariate ANOVA testing the influence of tall fescue cultivar, endophyte status and their interaction on total organic carbon and total phenolic content in root exudates**.

**Effect**	**Df_n,d_**	**mg C g^−1^ root**		**Phenolic content (μg ml^−1^g^−1^ root)**	
		**F**	**P**	**F**	**P**
Cultivar	1,1	0.06	0.81	5.03	**0.03**
Endophyte	3,3	8.30	**0.0006**	8.66	**0.0003**
Cultivar × Endophyte	3,3	4.09	**0.02**	3.75	**0.02**

*Significant effects (P ≤ 0.05) are shown in bold*.

### Identification and classification of metabolites via GC-(TOF) MS

**Figure 3:**
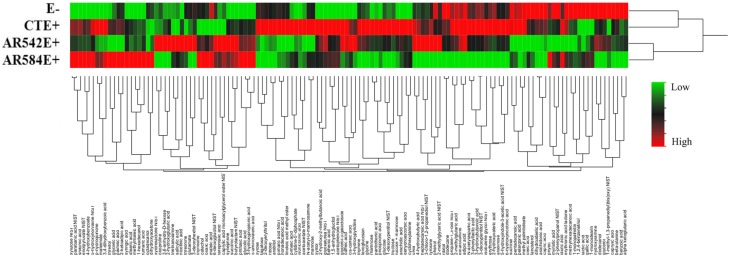
**Hierarchical clustering of root exudate compounds influenced by endophyte status (Ward's minimum variance method)**. The rows of the heat map represent different endophyte statuses (E-, endophyte free; CTE+, common toxic endophyte infected; AR542E+ and AR584E+, novel endophyte infected) while columns show the 132 root exudate compounds. The colors from green, black to red reflect the relative abundance of the metabolites from lowest to highest.

Over 300 peaks were present in the untargeted metabolomics GC-TOF-MS spectra of the root exudate samples. Processing of the raw data using the Binbase algorithm (Skogerson et al., 2011) resulted in the positive identification of 132 of these compounds (Table S3). The compounds identified were categorized into the following groups: sugars, polyols, growth factors and vitamins, lipids, amines, phenolics, carboxylic acids, nucleosides and others (Table S1). Of the 132 exudates, the levels of 43 compounds (excluding those classified as other) were significantly affected by tall fescue cultivar, endophyte status or cultivar × endophyte interaction (Table S2). Dendrograms obtained from cluster analysis of the 132 identified compounds by endophyte status (Figure 3) showed that root exudate profiles of E- and CTE+ were grouped and different from the composition of the two novel endophytes, which grouped together.

Sugars, growth factors and amines were significantly influenced by endophyte status, while the cultivar effects on amines, growth factors and others were significant (Table 3). Many individual compounds were also significantly affected by endophyte status, such as glucose, ribose, 6-hydroxynicotinic acid, myristic acid, pentadecanoic acid, phenol, 3-hydroxypropionic acid, and benzoic acid (Table S2).

**Table 3:** **Univariate ANOVA testing for the influence of tall fescue cultivar, endophyte status and their interaction on the normalized abundance of root exudate compounds by chemical category**.

**Compound class**	**Endophyte status**			**Tall fescue cultivar**			**Interaction**		
	**Df_n,d_**	**F**	**P**	**Df_n,d_**	**F**	**P**	**Df_n,d_**	**F**	**P**
Amines	3,3	10.10	**0.0008**	1,1	5.82	**0.03**	3,3	5.00	**0.01**
Carboxylic acids	3,3	0.95	0.45	1,1	0.23	0.64	3,3	0.88	0.47
Growth factors and vitamins	3,3	3.46	**0.05**	1,1	7.90	**0.01**	3,3	0.40	0.75
Lipids	3,3	1.16	0.36	1,1	1.02	0.33	3,3	3.25	**0.05**
Nucleic acids	3,3	0.63	0.61	1,1	1.99	0.18	3,3	4.05	**0.03**
Others	3,3	2.65	0.09	1,1	5.12	**0.04**	3,3	0.39	0.76
Phenolics	3,3	0.84	0.49	1,1	0.59	0.45	3,3	6.78	**0.005**
Polyols	3,3	1.23	0.34	1,1	0	0.99	3,3	4.77	**0.02**
Sugars	3,3	3.38	**0.05**	1,1	0.90	0.36	3,3	0.97	0.44

*Significant effects (P ≤ 0.05) are shown in bold*.

**Figure 4:**
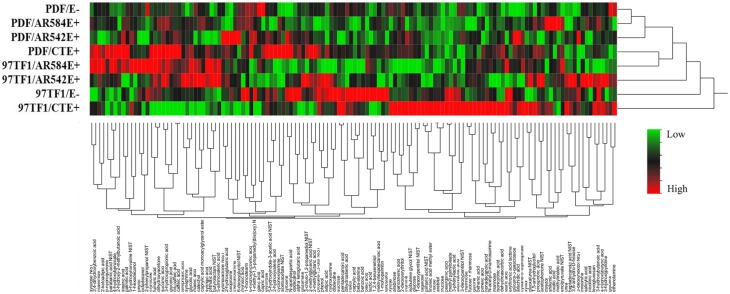
**Hierarchical cluster analysis of root exudate compounds influenced by tall fescue cultivar × endophyte status**. The rows of the heat map represent different endophyte statuses and tall fescue cultivar combinations and columns show the 132 root exudate compounds. The colors from green, black to red reflect the relative abundance of the metabolites from lowest to highest.

**Figure 5:**
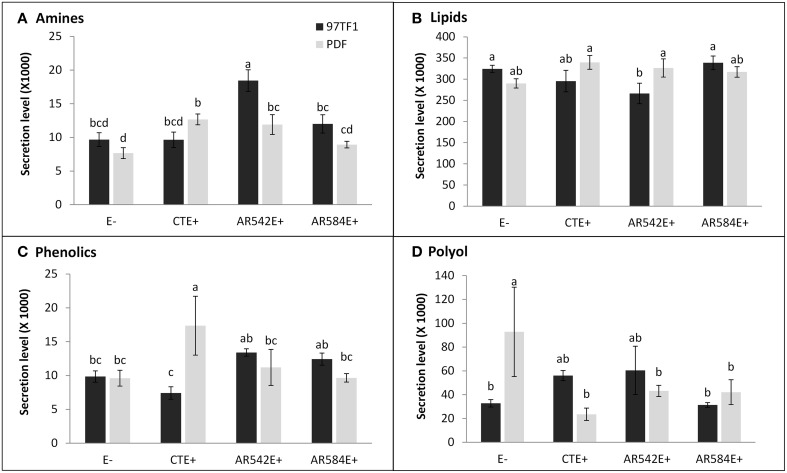
**Influence of tall fescue cultivar by endophyte interaction on the secretion level of root exudate components**. **(A)** Amines, **(B)** lipids, **(C)** phenolics, **(D)** polyol. Different letters indicate significant differences (Student's *t*-test, *P* ≤ 0.05) across endophyte statuses and tall fescue cultivar combinations.

Hierarchical analysis of 132 compounds including both cultivar and endophyte status revealed that PDF and 97TF1 tall fescue nearly formed two distinct groups (Figure 4). Within PDF, E- and AR584E+ were grouped together and were similar to AR542E+. PDF/CTE+, however, was grouped with 97TF1/AR584E+, while 97TF1/E-, and /CTE+ differed substantially from all the other cultivar/endophyte combinations. Secretion levels of exudate groups were influenced by cultivar and endophyte interactions (Table S4). For example, 97TF1/AR542E+ individuals exuded the highest level of amines while PDF/E- released only half the amount (Table S4 and Figure 5A). The secretion level for lipids from the 97TF1/AR542E+ combination was significantly lower than the 97TF1/E−, 97TF1/AR584E+, PDF/CTE+, and PDF/AR542E+ combinations (Table S4 and Figure 5B). PDF/CTE+ exuded significantly higher levels of phenolics than the other combinations (Table S4 and Figure 5C). The abundance of polyols was highest in PDF/E- samples and lower in all the endophyte infected PDF individuals, but differences across endophyte statuses for 97TF1 were less pronounced (Table S4 and Figure 5D). PDF/CTE+ and 97TF1/AR584E+ samples contained highest level of nucleic acid (Table S4). Twenty-six of the compounds in the exudate groups (excluding those listed as other) were found to be significantly impacted by the cultivar and endophyte interaction (Table S2) including arabinose, dihydroxyacetone, palmitic acid, caffeic acid, syringic acid, and terephtalic acid.

## Discussion

### Endophyte status and fescue cultivar affect plant biomass

Overall our results support the hypothesis that biomass production would be significantly influenced by the interaction between fungal endophyte status and tall fescue cultivar. Our results are consistent with those from several studies reporting endophyte—associated increases in plant biomass production in CTE+ infected vs. E- grasses (Arachevaleta et al., 1989; Debattista et al., 1990a). For example, Arachevaleta et al. (1989) using Kentucky-31 CTE+/E- clone pairs, found CTE+ clones produced slightly more dry herbage than E- clones under normal growth conditions, but produce up to 50% higher dry herbage biomass than their E- clones when grown with higher availability of N (Arachevaleta et al., 1989). Similarly, using six CTE+/E- clone pairs, De Battista et al. (1990) observed an overall increase in biomass production in CTE+ clones relative to the E- (Debattista et al., 1990b). Belesky and Fedders (1995) also observed an overall 15 and 11% increase in shoot and root biomass, respectively, in CTE+ vs. E- clones (Belesky and Fedders, 1995).

Under the controlled conditions of this study, novel endophyte AR542E+ did not perform as well as CTE+, from a biomass production perspective, particularly in cultivar PDF. This result is similar to that of Bouton et al. (2002) who found lower dry matter yield of AR542E+ infected Georgia 5 compare to the Georgia 5/CTE+ combination. Rudgers et al. (2010) also reported that Georgia 5/AR542E+ had lower biomass production than Georgia 5/CTE+, although the difference was not significant. Similar to our results, in a field study, PDF/AR542E+ also produced significantly lower forage yield than PDF/AR584E+, and lower yield than 97TF1/AR542E+ and 97TF1/AR584E+ combinations (Hopkins et al., 2010).

In general in our study, cultivar 97TF1 had higher total and shoot biomass production than PDF, though these cultivar differences were minimized in E- and AR584E+ material, but PDF had greater root:shoot ratios than 97TF1. Studies by (De Battista et al., 1990) and Belesky and Fedders (1995) also showed that plant biomass production depended on the tall fescue cultivar. Notably, Hill et al. (1990) observed a similar response wherein biomass production was influenced by both cultivar and endophyte presence and strain and concluded that the phenotypic variability brought about by infecting different plant genotypes with the same endophyte may introduce sufficient plasticity into a population such that the population as a whole is more adaptable to diverse environmental conditions. Introducing individuals with different strains of endophytes into the population may further increase population-level environmental plasticity.

The specific mechanisms responsible for endophyte-mediated alteration of root and shoot biomass and the interaction with tall fescue cultivar remain elusive. Several studies observed production of the growth hormone auxin (IAA) by *Balansia epichloë* (Porter et al., 1985) and by *Epichloë coenophiala* in tall fescue (De Battista et al., 1990; Tan and Zou, 2001). The degradation of host plant cell wall carbohydrates by endophytes (*Acremonium typhinum* and *Neotyphodium lolii*) to obtain carbon (Lam et al., 1994; Rasmussen et al., 2012), supported by the production of β-1,6-glucanase in *Epichloe festucae* and *Neotyphodium lolii* (Bryant et al., 2007), could be a possible mechanism for reduction in plant biomass. Complex interactions between the endophyte and the host plant genotype, including growth hormone regulation and fungal C utilization among others, most likely influence host plant growth responses, and may account for the diversity of endophyte and cultivar effects on biomass production reported in the literature.

### Endophyte status and fescue cultivar effect root exudate composition

Along with the phenotypic changes in response to endophyte status and tall fescue cultivar, rhizodeposit quantity and composition were also affected by the grass-endophyte symbiosis. Infection with CTE+ in cultivar 97TF1 resulted in the release of greater amounts of exudate total C (Figure 2A) compared to the other combinations. Greater total carbon content in root exudates from endophyte infected tall fescue has been attributed to higher photosynthetic rates presumably in response to higher maintenance costs of the symbiosis (Marks and Clay, 1996). Plants can release anywhere from 5 to 10% of their fixed C into the rhizosphere; however, in hydroponics the amount is likely underestimated because the same concentration gradient across the cell wall does not exist due to C not being consumed by soil organisms or rendered unavailable due to sorption to soil constituents (Personeni et al., 2007). Therefore, it is possible that our experimental conditions resulted in a dampened effect compared to what might be observed in a true soil system. We are exploring this possibility in a complementary greenhouse rhizobox study; however, since it is well known that a multitude of plant-soil interactions influence root exudate production (Uren, 2007), this pure culture test highlights cultivar-endophyte-rhizodeposit effects which should aid in interpretation of a more complex system.

Endophyte-associated increased exudation of C is important from the perspective of C sequestration. Carbon released from plant roots, depending on the chemical form, can be rapidly mineralized (0.5–2 h) by the rhizosphere microbial community, and/or incorporated into microbial biomass where it can persist for longer time periods (30–90 days) (Jones et al., 2004). Several studies have reported increases in C and N in pasture soils throughout the southeastern United States under CTE+ compared to E- infected tall fescue (Franzluebbers et al., 1999b; Franzluebbers and Stuedemann, 2005; Iqbal et al., 2012), although in other studies the same trend was not observed (Handayani et al., 2011). If endophyte-associated changes to root exudation mediate the observed alterations to total C and N pools, the fact that we observed increased total C exudation in CTE+/97TF1 but not CTE+/PDF suggests that the conflicting reports may be related to different cultivars being examined. The interaction of endophyte status and tall fescue cultivar on the amount of rhizodeposit C exuded and root biomass produced, and how these parameters interact with the many edaphic factors present in a natural setting, may help explain these inconsistencies (Franzluebbers and Stuedemann, 2002, 2005; Franzluebbers, 2006; Iqbal et al., 2012).

Not only does the total quantity of C in root exudates affect soil nutrient pools, but also the composition. Chemical composition of the exudates can influence microbial community structure, function and other important biogeochemical processes within the rhizosphere ultimately influencing nutrient cycling at a larger scale. This is the first paper to report on the detailed composition of root exudates from different tall fescue cultivars and how they are influenced by endophyte infection status. Several of the root exudate compounds identified have previously been shown to influence nutrient acquisition, cause shifts in soil microbial community structure and have been linked to allelopathy (Hoffland et al., 1992; Dakora and Phillips, 2002; Bais et al., 2006; Shi et al., 2011). Endophyte-associated changes in exudate composition may provide a potential mechanism explaining how the tall fescue—endophyte symbiosis alters these processes.

Previous studies found that endophyte infection increases the exudation of phenolics and postulated that they may be involved in the acquisition of P from soils (Malinowski et al., 1998; McNear and McCulley, 2012). Phenolic compounds and carboxylic acids can increase P availability by increasing Fe^3+^ reducing activity in the rhizosphere, forming complexes with Al and Fe, and/or competing with P for binding sites on clay and humus colloids (Lipton et al., 1987; Hoffland et al., 1992; Jones, 1998; Malinowski et al., 1998, 2004; Malinowski and Belesky, 1999b). In this study, endophyte status and its interaction with tall fescue cultivar (especially 97TF1/CTE+) significantly increased total phenolic content detected by colorimetric methods (Figure 2B) and influenced the release of specific carboxylic acids and phenolics (Table S2) shown to be involved in P acquisition. Notably, results from the colorimetric method for determining total phenolic content were different from the total phenolic content determined by summing all the identified phenolics using GC-MS. This likely occurred because only 132 of over 300 peaks (i.e., chemicals) within the exudates were positively identified using GC-MS, some of which are likely yet-to-be identified phenolics which may have yet-to-be identified rhizosphere functions.

Endophyte status and its interaction with tall fescue cultivar also influenced the release of specific carboxylic acids and phenolics (Table S2) shown to be involved in P acquisition. Succinic acid levels were two times higher in novel endophyte infected tall fescue (data not shown) compared to that of CTE+ and E- plants. Enhanced exudation of succinic acid has been observed from the roots of tomato plants grown under low phosphorus condition (Imas et al., 1997). Overall, infection with CTE+ resulted in a significant increase in glucose (data not shown) compared to other endophytes, which have been shown to increase organic P mineralization by stimulating the microbial community and phosphatase activity (Falih and Wainwright, 1996; Hamel, 2004; Spohn et al., 2013). More research is still needed to clarify the mechanisms behind how these exudate compounds influence nutrient acquisition under more realistic growth conditions. However, these results provide evidence that root exudates associated with nutrient acquisition are influenced by endophyte status and its interaction with its host plant.

Root exudates can also influence plant community structure either indirectly by altering biogeochemical processes within the rhizosphere or directly by inhibiting seed germination or growth of neighboring plants (Asao et al., 2003; Kalinova et al., 2007). Rudgers et al. (2010) reported that plots containing Kentucky-31 tall fescue infected with CTE+ had lower plant diversity, specifically lower graminoid and forb biomass, than plots growing Georgia-5 tall fescue harboring the novel endophyte AR542E+. Similarly, Georgia-5/CTE+ was found to have a lower proportion of forbs compared to fields growing Georgia-5/AR542E+ (Yurkonis et al., 2014). The release of chemicals unique to CTE+ tall fescue, and possibly allelopathic, could be a mechanism contributing to the observed endophtye effects on plant succession and whole ecosystem biodiversity in these studies.

For example, syringic acid, a phenolic, was significantly influenced by the interaction of cultivar and endophyte in this study (highest in 97TF1/AR584E+, lowest in PDF/AR542E+), and syringic acid in black mustard root exudates has been shown to inhibit germination and growth of two weeds, *Phalaris paradoxa* and *Sisymbrium irio* (Al-Sherif et al., 2013). Similarly, levels of the lipid myristic acid were significantly different between AR542E+ and AR584E+ (AR542E+<AR584E+), and myristic acid in root exudates of cucumber was shown to inhibit the growth of lettuce (Yu and Matsui, 1994); However, endophyte presence did not lead to greater production of all growth inhibiting chemicals. For example, endophyte presence resulted in reduced production of benzoic acid (Table S2) relative to E- plants which has been found to be allelopathic to lettuce (Yu and Matsui, 1994) and soybean (Baziramakenga et al., 1995). CTE+ infected tall fescue has also been shown to have nematocidal properties which could indirectly improve plant productivity by inhibiting nematode infestation (West et al., 1988; Elmi et al., 2000). We found that palmitic acid, a lipid, was significantly lower in AR542E+ tall fescue than CTE+ and AR584E+, and palmitic acid has been shown to significantly reduced *Meloidogyne incognita* (root-knot nematodes) reproduction. It is possible that endophyte and cultivar associated changes in the production of such compounds may lead to community- and ecosystem-level effects.

Rhizodeposits together with root system architecture play an integral part in shaping the rhizosphere microbial community. The expectation is, given the same soil type, if two plants have different root biomass, architecture and exudate profiles, they would select for different rhizosphere microbial communities (Jenkins et al., 2006; Mack and Rudgers, 2008; Casas et al., 2011). Therefore, if leaf fungal endophytes in tall fescue alter root biomass and exudate composition, as we have shown here, we hypothesize that this could have an influence on rhizosphere microbial community structure and/or function. Data on the influence of endophyte infection on rhizosphere microbial communities are inconsistent (Omacini et al., 2012), although several studies have shown soils under CTE+ infected tall fescue to have different microbial community structure and/or function relative to those under endophyte-free plants (Jenkins et al., 2006; Buyer et al., 2011; Iqbal et al., 2012). Van Hecke et al. (2005) reported no detectable changes in microbial community structure (using culture based methods) in soils under CTE+ infected tall fescue cultivar “Jessup” relative to its endophyte-free isogenic line but did find increased soil respiration rates which they hypothesized had to do with greater total carbon and sugar content in E+ rhizodeposits (Van Hecke et al., 2005). In contrast, CTE+ tall fescue was shown to increase *Cytophaga-Flavobacterial* cell counts in rhizosphere soil which may be related to their capacity to degrade macro-molecules (e.g., polysaccharides) which we found to be highest in exudates of CTE+ tall fescue plants (Hans, 2006; Jenkins et al., 2006).

Sugars are typically the most abundant rhizodeposits from plant roots and are utilized directly by microbes for growth (Griffiths et al., 1999; Landi et al., 2006; Eilers et al., 2010). Glucose has been shown to influence the soil microbial community in a variety of ways (Falchini et al., 2003; Landi et al., 2006). According to Jones et al. (2003), sugars and amines are used for microbial growth while organic acids are used primarily for respiration (Jones et al., 2003). Chaparro et al. (2013) hypothesized that more sugars are released early in plant development to stimulate microbial colonization of the rhizosphere, but as the plant develops, the proportion of sugars decreases and more phenolics and amino acids are released that act to select for less diverse, but more specialized microorganisms (i.e., microbes effective against root pathogens) (Chaparro et al., 2013). In this study, total sugar content was higher in CTE+ infected tall fescue (notably in 97TF1), and arabinose, glucose, levoglucosan and ribose were significantly higher in CTE+ compare to endophyte free tall fescue and novel endophyte infected plant root exudates (data not shown). These results suggest that infection with CTE+, especially in certain cultivars such at 97TF1, could have a significant impact on soil microbial community structure via increased sugar exudation. Similarly, significantly greater secretion levels of amines were observed in PDF/AR542E+ compared to PDF/AR584E+ infected individuals (Figure 5A). Amines can function as C and N sources for microbes and are involved in microbial colonization of the rhizosphere and nitrogen cycling (Simons et al., 1997; De Nobili et al., 2001; Oku et al., 2012; Moe, 2013). Such nitrogen containing inputs, once mineralized, may induce significantly higher fluxes of trace gas from the soil (McLain and Martens, 2005) possibly explaining the observations of greater CO_2_ and N_2_O fluxes under PDF/AR542E+ stands (Iqbal et al., 2013). Overall, these results indicate that endophyte strains interacting with host cultivar could be involved in changing microbial growth, metabolism and soil organic matter turnover via rhizosphere priming which could eventually alter C and N storage in CTE+ infected tall fescue pastures throughout the Southeastern United States (Kuzyakov, 2002; Hamer and Marschner, 2005).

## Conclusion

We have shown that endophyte status, tall fescue cultivar and their interaction can have a significant influence on early stages of plant growth, root exudate composition and quantity. This study revealed that root exudate compounds released by tall fescue contain a rich chemical diversity, including sugars, phenolics, lipids and carboxylic acids, and also found that root exudate composition is often specific to the endophyte and cultivar combination being examined. These findings illustrate that aboveground fungal endophytes can alter root exudate composition that may then influence soil biogeochemical processes (e.g., carbon and nitrogen storage, soil microbial community structure and function, enzyme activity). While these studies provide an indication of the potential for endophyte and tall fescue cultivar mediated changes in soil biogeochemical processes, we acknowledge that the experiments were conducted under controlled environmental conditions and do not reflect the complex edaphic and climactic interactions found under more realistic field conditions which will likely alter the predicted response. We also only sampled at one time point during the plants development which doesn't reflect the changes in exudate composition that have been shown to occur as plants develop (Chaparro et al., 2013). It will be important to assess how the grass-fungus interaction changes exudate composition with plant development and across diverse environmental conditions. The sorption, degradation, and utilization of root exudates when these plants are grown in soils is certain to alter the effectiveness of many of the exudates identified here which may be why climate, stand age, site (i.e., soil type) and field management (e.g., grazing) were found to be some of the strongest predictors of C sequestration in the field (Iqbal et al., 2012). Even given the diversity of environmental controls on root exudation, these pure culture results clearly identify possible cultivar-endophyte-rhizodeposit effects and will help guide interpretation of likely more complex results obtained from rhizobox and field plot studies.

## Author contributions

DHMJ and RLM conceptualized and designed the experiments, and helped in the analysis and interpretation of the data. JG acquired, analyzed and helped in the interpretation of the data and drafted the manuscript. DHMJ and RLM contributed to critical revision of the manuscript.

### Conflict of interest statement

The authors declare that the research was conducted in the absence of any commercial or financial relationships that could be construed as a potential conflict of interest.
