# Supplementary material for: Tall fescue cultivar and fungal endophyte combinations influence plant growth and root exudate composition
Source: Front Plant Sci. 2015 Apr 9;6:183. doi: 10.3389/fpls.2015.00183 (PMC4391242; doi:10.3389/fpls.2015.00183)
Supplement: Supplementary file 1 [file Table1.DOCX]

**Table S1:** Classification of root exudate compounds identified by binbase

| Class of compounds |  | Class of compounds |  |
| --- | --- | --- | --- |
| Amines (amino acid, amino alcohol and etc) | 5-aminovaleric acid, 5-hydroxynorvaline NIST, β-alanine, cyclohexylamine, ethanolamine, glutamate, glycine, N-acetylaspartic acid, N-acetyl-D-mannosamine, oxoproline, synephrine, threonine, triethanolamine, tyrosine | **Carboxylic acids** | 2-hydroxy-2-methylbutanoic acid, 2-hydroxyglutaric acid, 2-hydroxyvaleric acid, 2-ketoadipic acid, 2-methyglutaric acid, 2- methyglyceric acid NIST, 3-hydroxybutanoic acid, 3-hydroxypropionic acid, 3-phenyllactic acid, 4-hydroxybuturic acid, acetoacetate NIST, adipic acid, alpha ketoglutaric acid, behenic acid, benzoic acid, enolpyruvate NIST, fumaric acid, glycolic acid, lactic acid, maleic acid, maleimide, methylmaleic acid, oxalic acid, phthalic acid, pimaric acid NIST, pimelic acid, shikimic acid, succinic acid, terephtalic acid |
| Lipids (fatty acids, fatty alcohol and etc) | 1-hexadecanol, 1-monopalmitin, 1-monostearin, 2-deoxyerythritol, arachidic acid, arachidonic acid, capric acid, caprylic acid,dodecanol, heptadecanoic acid NIST, lauric acid, methy palmitoleate, methylhexadecanoic acid, myristic acid, nonadecanoic acid, octadecanol, oleic acid, palmitic acid,pelargonic acid, pentadecanoic acid, stearic acid | **Phenolics** | 1,2,4-benzenetriol, 3,4-dihydroxybenzoic acid, 4-hydroxybenzoate, caffeic acid, catechol, cis-caffeic acid, gallic acid, phenol, p-hydroquinone, salicylic acid, syringic acid, |
|  |  | **Nucleosides** | Cytidine-5’-diphosphate, thymidine |
| Growth factors and vitamins | 5-hydroxyindole-3-acetic acid NIST, 6-hydroxynicotinic acid, dehydroascorbic acid, nicotinic acid, pantothenic acid | **Polyols/Sugar alcohols** | 1-desoxypentitol NIST, 3-deoxypentitol NIST, 6-deoxyglucitol NIST, arabitol, glycerol, glycerol-3-galactoside, ribitol, threitol, xylitol |
| Sugars | 1,5-anhydroglucitol, 3,6-anhydro-D-hexose, 3-deoxyhexitol NIST, arabinose, dihydroxyacetone, erythrose, fructose, fucose+rhamnose, glucose, glyceric acid, levoglucosan, mannose, rhamonse, ribose, sucrose, tagatose, xylose, xylulose NIST | **Others** | (1-methyl-1,3-propanediyl)bis(oxy) NIST, 2-phenylpropanol NIST, 3-chloro-1,2-propanediol NIST, 3-hydroxypyridine, acetophenone NIST, butyrolactam NIST, caprylic acid monoacylglycerol ester NIST, dihydroabietic acid, dodecane, erythronic acid lactone, glyoxalurea NIST, hydroxylamine, isobutene glycol NIST, isonicotinic acid, lanosterol, linoleic acid methyl ester, parabanic acid NIST, phosphoric acid, phytol, propane-1-3-diol NIST, urea, xylonolactone NIST |
