# Supplementary material for: Tall fescue cultivar and fungal endophyte combinations influence plant growth and root exudate composition
Source: Front Plant Sci. 2015 Apr 9;6:183. doi: 10.3389/fpls.2015.00183 (PMC4391242; doi:10.3389/fpls.2015.00183)
Supplement: Supplementary file 2 [file Table2.DOCX]

**Table S2**: ANOVA results testing normalized abundance of 43 root exudate compounds that were significantly influenced by tall fescue cultivar, endophyte status and/or the cultivar by endophyte interaction. Significant effects (P ≤ 0.05) are shown in bold.

|  |  | Endophyte status | | Tall Fescue cultivar | | |  | Interaction | |
| --- | --- | --- | --- | --- | --- | --- | --- | --- | --- |
|  | **df** | **F** | **P** | **df** | **F** | **P** | **df** | **F** | **P** |
| *Amines* |  |  |  |  |  |  |  |  |  |
| Glutamate | 3,3 | 3.5847 | **0.04** | 1,1 | 2.3047 | 0.15 | 3,3 | 3.1801 | 0.06 |
| Synephrine | 3,3 | 22.2373 | **<0.0001** | 1,1 | 8.2715 | **0.01** | 3,3 | 28.7455 | **<0.0001** |
| Thymine | 3,3 | 3.5525 | **0.04** | 1,1 | 0.2485 | 0.63 | 3,3 | 1.0072 | 0.42 |
| Triethanolamine | 3,3 | 3.9764 | **0.03** | 1,1 | 12.297 | **0.004** | 3,3 | 2.0787 | 0.15 |
| Tyrosine | 3,3 | 1.9433 | 0.17 | 1,1 | 0.0098 | 0.92 | 3,3 | 4.1072 | **0.03** |
| *Carboxylic Acids* |  |  |  |  |  |  |  |  |  |
| 2-Hydroxyvaleric Acid | 3,3 | 4.701 | **0.02** | 1,1 | 1.4631 | 0.25 | 3,3 | 2.6608 | 0.09 |
| 3-Hydroxypropionic Acid | 3,3 | 5.9366 | **0.008** | 1,1 | 1.9654 | 0.18 | 3,3 | 7.7158 | **0.003** |
| Behenic Acid | 3,3 | 0.5874 | 0.63 | 1,1 | 4.7054 | **0.05** | 3,3 | 0.8268 | 0.50 |
| Benzoic Acid | 3,3 | 5.7399 | **0.009** | 1,1 | 2.3281 | 0.15 | 3,3 | 3.0854 | 0.06 |
| Fumaric Acid | 3,3 | 4.2752 | **0.02** | 1,1 | 0.4221 | 0.53 | 3,3 | 25.1122 | **<0.0001** |
| Glycolic Acid | 3,3 | 9.3287 | **0.001** | 1,1 | 0.2118 | 0.65 | 3,3 | 15.2852 | **0.0001** |
| Lactic Acid | 3,3 | 4.8905 | **0.02** | 1,1 | 15.9707 | **0.001** | 3,3 | 12.8876 | **0.0003** |
| Maleic Acid | 3,3 | 1.2454 | 0.33 | 1,1 | 2.1302 | 0.17 | 3,3 | 4.7737 | **0.02** |
| Phthalic Acid | 3,3 | 2.9309 | 0.07 | 1,1 | 3.5941 | 0.08 | 3,3 | 3.7373 | **0.04** |
| Succinic Acid | 3,3 | 19.4509 | **<0.0001** | 1,1 | 16.8561 | **0.001** | 3,3 | 28.0531 | **<0.0001** |
| Terephtalic Acid | 3,3 | 44.9315 | **<0.0001** | 1,1 | 15.4507 | **0.002** | 3,3 | 37.0126 | **<0.0001** |
| *Growth Factor* |  |  |  |  |  |  |  |  |  |
| 6-Hydroxynicotinic Acid | 3,3 | 5.3199 | **0.01** | 1,1 | 13.4987 | **0.003** | 3,3 | 1.3522 | 0.30 |
| *Lipids* |  |  |  |  |  |  |  |  |  |
| 1-Monostearin | 3,3 | 2.4705 | **0.05** | 1,1 | 3.0769 | 0.10 | 3,3 | 2.3448 | 0.12 |
| Caprylic Acid | 3,3 | 4.4933 | **0.02** | 1,1 | 4.1803 | 0.06 | 3,3 | 0.5569 | 0.65 |
| Myristic Acid | 3,3 | 6.7837 | **0.005** | 1,1 | 2.0402 | 0.18 | 3,3 | 2.2512 | 0.13 |
| Palmitic Acid | 3,3 | 6.6462 | **0.005** | 1,1 | 17.3826 | **0.0009** | 3,3 | 4.5861 | **0.02** |
| Pentadecanoic Acid | 3,3 | 4.4268 | **0.02** | 1,1 | 1.1599 | 0.30 | 3,3 | 0.9978 | 0.42 |
| *Phenolics* |  |  |  |  |  |  |  |  |  |
| 4-Hydroxybenzoate | 3,3 | 1.578 | 0.24 | 1,1 | 0.0003 | 0.99 | 3,3 | 3.6449 | **0.04** |
| Caffeic Acid | 3,3 | 2.2978 | 0.12 | 1,1 | 2.3266 | 0.15 | 3,3 | 15.4004 | **0.0001** |
| Cis-Caffeic Acid | 3,3 | 2.353 | 0.12 | 1,1 | 2.8561 | 0.11 | 3,3 | 14.7132 | **0.0001** |
| Phenol | 3,3 | 5.4703 | **0.01** | 1,1 | 4.1191 | 0.06 | 3,3 | 1.4088 | 0.28 |
| Syringic Acid | 3,3 | 1.5316 | 0.25 | 1,1 | 0.4337 | 0.52 | 3,3 | 3.9015 | **0.03** |
| *Polyols* |  |  |  |  |  |  |  |  |  |
| 1-Desoxypentitol NIST | 3,3 | 8.7542 | **0.002** | 1,1 | 1.6876 | 0.21 | 3,3 | 3.7874 | **0.04** |
| 3-Deoxypentitol NIST | 3,3 | 2.6994 | 0.09 | 1,1 | 0.0609 | 0.81 | 3,3 | 4.6677 | **0.02** |
| 6-Deoxyglucitol NIST | 3,3 | 4.9841 | **0.01** | 1,1 | 2.5101 | 0.14 | 3,3 | 0.2219 | 0.88 |
| Glycerol | 3,3 | 1.7668 | 0.20 | 1,1 | 0.2637 | 0.62 | 3,3 | 5.2293 | **0.01** |
| Xylitol | 3,3 | 2.1321 | 0.14 | 1,1 | 4.6847 | **0.05** | 3,3 | 1.4488 | 0.27 |
| *Sugars* |  |  |  |  |  |  |  |  |  |
| 3-Deoxyhexitol NIST | 3,3 | 1.1193 | **0.02** | 1,1 | 1.5965 | 0.23 | 3,3 | 8.7263 | **0.002** |
| Arabinose | 3,3 | 10.852 | **0.0006** | 1,1 | 0.1636 | 0.69 | 3,3 | 10.9837 | **0.0006** |
| Dihydroxyacetone | 3,3 | 1.5053 | 0.26 | 1,1 | 0.2142 | 0.65 | 3,3 | 4.6572 | **0.02** |
| Fructose | 3,3 | 3.0087 | 0.07 | 1,1 | 6.3028 | **0.03** | 3,3 | 1.5789 | 0.24 |
| Glucose | 3,3 | 6.6529 | **0.005** | 1,1 | 1.9446 | 0.18 | 3,3 | 3.0158 | 0.07 |
| Glyceric Acid | 3,3 | 2.4493 | 0.11 | 1,1 | 19.4749 | **0.0006** | 3,3 | 10.897 | **0.0006** |
| Levoglucosan | 3,3 | 11.2524 | **0.0005** | 1,1 | 0.7424 | 0.40 | 3,3 | 18.1618 | **<0.0001** |
| Ribose | 3,3 | 11.3741 | **0.0005** | 1,1 | 15.5576 | **0.001** | 3,3 | 8.0684 | **0.002** |
| Tagatose | 3,3 | 3.5032 | **0.04** | 1,1 | 2.0894 | 0.17 | 3,3 | 2.27 | 0.12 |
| *Nucleosides* |  |  |  |  |  |  |  |  |  |
| Cytidine-5'-Diphosphate | 3,3 | 3.0137 | 0.07 | 1,1 | 2.3409 | 0.15 | 3,3 | 5.0243 | **0.01** |
| Thymidine | 3,3 | 0.4557 | 0.72 | 1,1 | 0.0226 | 0.88 | 3,3 | 4.6749 | **0.02** |
