# Supplementary material for: Tall fescue cultivar and fungal endophyte combinations influence plant growth and root exudate composition
Source: Front Plant Sci. 2015 Apr 9;6:183. doi: 10.3389/fpls.2015.00183 (PMC4391242; doi:10.3389/fpls.2015.00183)
Supplement: Supplementary file 3 [file Table3.DOCX]

**Table S3** Secretion level of root exudate compounds released by different endophyte strains average across tall fescue cultivars identified by GC-TOF MS. Numbers indicate the average peak height of three replicates in each endophyte status. Numbers in parenthesis indicate the standard error.

|  | E- | CTE+ | AR542E+ | AR584E+ |
| --- | --- | --- | --- | --- |
| 1,2,4-benzenetriol | 176.00 (14.27) | 160.17 (11.68) | 162.67 (18.95) | 163.50 (9.06) |
| 1,5-anhydroglucitol | 370.50 (36.22) | 1047.34 (198.52) | 634.17 (88.43) | 771.50 (176.78) |
| 1-desoxypentitol NIST | 329.33 (55.58) | 1085.67 (529.98) | 498.17 (127.61) | 271.34 (59.13) |
| 1-hexadecanol | 1105.84 (208.32) | 1245.00 (297.13) | 1231.17 (220.69) | 2198.84 (589.18) |
| 1-methyl-1,3-propanediyl)bis(oxy) NIST | 10383.00 (2001.19) | 4879.50 (558.67) | 7826.17 (3436.01) | 5462.00 (909.79) |
| 1-monopalmitin | 185.34 (23.76) | 156.34 (13.26) | 148.00 (15.69) | 154.67 (11.40) |
| 1-monostearin | 148.50 (9.35) | 90.84 (18.39) | 111.84 (15.76) | 125.67 (10.92) |
| 2-deoxyerythritol | 1997.17 (558.81) | 2786.00 (921.30) | 1620.17 (307.56) | 1565.67 (519.81) |
| 2-hydroxy-2-methylbutanoic acid | 617.67 (53.14) | 965.34 (302.88) | 886.00 (136.87) | 777.00 (130.68) |
| 2-hydroxyglutaric acid | 481.17 (47.60) | 521.17 (101.60) | 610.00 (54.85) | 471.00 (52.70) |
| 2-hydroxyvaleric acid | 1765.67 (135.99) | 1937.67 (349.80) | 1338.34 (170.56) | 1105.17 (79.08) |
| 2-ketoadipic acid | 1251.34 (82.13) | 1356.67 (151.92) | 1108.17 (110.99) | 1490.00 (226.57) |
| 2-methylglutaric acid | 148.67 (24.16) | 152.17 (28.58) | 138.34 (33.04) | 96.17 (12.71) |
| 2-methylglyceric acid NIST | 215.34 (25.88) | 212.67 (33.98) | 232.17 (32.42) | 185.00 (22.34) |
| 2-phenylpropanol NIST | 548.34 (30.32) | 436.17 (34.64) | 358.00 (23.16) | 464.00 (79.24) |
| 3,4-dihydroxybenzoic acid | 1517.00 (128.65) | 2040.67 (454.37) | 1557.67 (92.42) | 2023.00 (220.66) |
| 3,6-anhydro-D-hexose | 168.84 (31.66) | 181.00 (22.41) | 259.50 (82.38) | 164.50 (13.94) |
| 3-chloro-1,2-propanediol NIST | 511.17 (47.38) | 742.67 (202.13) | 1048.50 (370.84) | 363.17 (43.17) |
| 3-deoxyhexitol NIST | 298.84 (71.46) | 255.34 (74.99) | 395.17 (61.44) | 201.00 (26.98) |
| 3-deoxypentitol NIST | 446.34 (30.59) | 512.34 (271.13) | 371.67 (114.84) | 218.50 (27.72) |
| 3-hydroxybutanoic acid | 455.00 (91.18) | 476.00 (80.27) | 427.17 (85.63) | 354.84 (98.67) |
| 3-hydroxypropionic acid | 995.50 (160.75) | 1479.34 (465.70) | 1870.17 (280.37) | 2203.84 (303.31) |
| 3-hydroxypyridine | 1722.67 (335.22) | 2037.00 (222.66) | 2231.67 (260.71) | 1763.84 (121.41) |
| 3-phenyllactic acid | 444.84 (98.04) | 527.34 (184.40) | 405.67 (74.94) | 298.17 (48.45) |
| 4-hydroxybenzoate | 715.34 (99.08) | 932.00 (177.94) | 805.00 (56.53) | 1035.67 (120.01) |
| 4-hydroxybutyric acid | 281.84 (26.23) | 400.17 (90.51) | 445.00 (91.84) | 220.34 (58.72) |
| 5-aminovaleric acid | 357.00 (104.28) | 424.67 (75.13) | 495.00 (135.73) | 521.67 (118.18) |
| 5-hydroxyindole-3-acetic acid NIST | 252.17 (41.63) | 319.84 (87.43) | 243.34 (61.67) | 196.00 (39.01) |
| 5-hydroxynorvaline NIST | 629.67 (195.26) | 930.67 (117.27) | 805.67 (171.29) | 943.50 (196.89) |
| 6-deoxyglucitol NIST | 12241.67 (2169.25) | 7837.17 (1551.97) | 14413.67 (1435.13) | 12792.84 (1582.04) |
| 6-hydroxynicotinic acid | 3111.17 (899.87) | 4193.00 (1326.11) | 2642.50 (1212.22) | 1879.50 (1657.97) |
| Acetoacetate NIST | 230.84 (25.54) | 589.84 (258.20) | 217.00 (35.08) | 247.34 (63.47) |
| Acetophenone NIST | 812.84 (77.71) | 1053.34 (109.82) | 971.34 (208.01) | 982.84 (94.76) |
| Adipic acid | 615.67 (63.84) | 615.50 (105.11) | 615.50 (80.04) | 504.00 (56.26) |
| Alpha ketoglutaric acid | 173.50 (16.00) | 154.84 (16.34) | 156.50 (15.28) | 148.84 (14.97) |
| Arabinose | 1212.84 (282.56) | 2998.84 (1053.57) | 1171.17 (177.30) | 578.17 (118.65) |
| Arabitol | 151.67 (18.41) | 302.50 (187.04) | 122.17 (12.86) | 112.00 (8.90) |
| Arachidic acid | 2429.34 (200.38) | 2972.50 (167.23) | 2739.84 (273.95) | 2561.67 (215.64) |
| Arachidonic acid | 682.00 (80.09) | 616.34 (75.23) | 524.34 (63.52) | 522.34 (60.53) |
| Behenic acid | 717.17 (80.46) | 992.84 (283.04) | 864.50 (37.36) | 897.17 (70.67) |
| Benzoic acid | 28934.50 (2106.23) | 23155.17 (758.52) | 23867.00 (1522.2) | 22309.34 (734.49) |
| Beta-alanine | 162.00 (13.64) | 148.84 (9.70) | 141.50 (16.84) | 132.67 (4.95) |
| Butyrolactam NIST | 537.67 (85.68) | 647.17 (66.37) | 854.50 (180.47) | 819.34 (108.36) |
| Caffeic acid | 2039.67 (326.06) | 3834.17 (2188.00) | 3707.00 (916.50) | 2475.17 (377.90) |
| Capric acid | 1688.17 (260.85) | 1589.00 (178.70) | 1386.84 (109.15) | 1360.17 (174.91) |
| Caprylic acid | 1431.50(129.87) | 1177.00 (60.79) | 1113.00 (62.38) | 1035.00 (42.57) |
| Caprylic acid monoacylglycerol ester NIST | 185.84 (30.21) | 166.84 (27.36) | 264.00 (94.75) | 246.17 (55.10) |
| Catechol | 317.84 (16.20) | 282.84 (24.01) | 330.50 (28.51) | 348.50 (50.39) |
| Cis-caffeic acid | 697.50 (105.70) | 1049.00 (454.03) | 1171.00 (297.72) | 883.67 (117.20) |
| Cyclohexylamine | 177.50 (16.34) | 289.50 (89.42) | 212.17 (45.01) | 223.17 (76.22) |
| Cytidine-5'-diphosphate | 233.34 (24.16) | 318.17 (18.29) | 242.50 (38.66) | 265.17 (33.62) |
| Dehydroascorbic acid | 282.50 (41.97) | 632.34 (153.97) | 384.00 (86.15) | 383.17 (108.65) |
| Dihydroabietic acid | 242.50 (20.42) | 215.67 (22.23) | 177.17 (15.64) | 177.50 (12.58) |
| Dihydroxyacetone | 233.34 (25.66) | 220.50 (44.65) | 230.00 (25.16) | 282.67 (25.25) |
| Dodecane | 447.50 (68.99) | 395.67 (21.83) | 363.84 (43.26) | 283.50 (45.45) |
| Dodecanol | 736.50 (83.99) | 651.17 (64.84) | 696.17 (104.92) | 559.17 (41.37) |
| Enolpyruvate NIST | 196.00 (12.10) | 222.00 (32.66) | 208.34 (14.11) | 239.17 (27.62) |
| Erythronic acid lactone | 1909.34 (292.37) | 1892.17 (514.96) | 1741.67 (322.62) | 1869.67 (230.86) |
| Erythrose | 1370.84 (255.43) | 1691.17 (427.47) | 978.50 (379.06) | 715.84 (169.61) |
| Ethanolamine | 1132.34 (348.90) | 1208.67 (347.83) | 1166.34 (243.15) | 548.67 (44.46) |
| Fructose | 156.84 (30.14) | 536.67 (253.32) | 165.00 (23.48) | 120.84 (7.24) |
| Fucose + rhamnose | 1144.50 (224.98) | 1939.00 (624.30) | 1462.34 (144.92) | 1183.84 (233.00) |
| Fumaric acid | 432.00 (75.78) | 477.17 (164.03) | 492.50 (141.59) | 653.50 (95.09) |
| Gallic acid | 466.50 (81.53) | 312.17 (24.84) | 334.17 (43.09) | 399.17 (43.38) |
| Glucose | 210.67 (20.34) | 376.34 (73.52) | 236.00 (15.96) | 175.84 (22.35) |
| Glutamate | 303.00 (45.40) | 231.00 (42.93) | 500.50 (150.52) | 200.00 (24.30) |
| Glyceric acid | 701.84 (176.60) | 816.17 (318.12) | 654.00 (100.86) | 955.84 (198.52) |
| Glycerol | 47017.34 (22228.06) | 25373.00 (6700.34) | 33800.00 (9504.35) | 17658.67 (2802.22) |
| Glycerol-3-galactoside | 361.17 (50.51) | 513.84 (138.55) | 514.67 (202.17) | 381.00 (64.44) |
| Glycine | 178.17 (18.54) | 231.84 (54.60) | 202.17 (32.38) | 165.50 (16.66) |
| Glycolic acid | 1531.50 (257.23) | 2372.17 (842.70) | 3239.84 (924.12) | 3976.84 (645.91) |
| Glyoxalurea NIST | 566.84 (112.48) | 1310.84 (196.15) | 1237.84 (304.60) | 1063.50 (151.79) |
| Heptadecanoic acid NIST | 798.00 (149.66) | 877.17 (102.93) | 1025.67 (297.16) | 664.67 (72.81) |
| Hydroxylamine | 9568.84 (1043.31) | 7278.67 (1067.71) | 8892.67 (717.67) | 5792.00 (614.20) |
| Isobutene glycol NIST | 249.84 (30.00) | 306.00 (51.87) | 257.50 (18.47) | 171.67 (13.53) |
| Isonicotinic acid | 228.50 (29.75) | 580.50 (97.19) | 244.34 (25.41) | 244.50 (19.11) |
| Lactic acid | 2439.34 (761.15) | 1303.17 (166.02) | 1305.67 (150.25) | 1746.00 (169.91) |
| Lanosterol | 197.67 (16.12) | 172.84 (12.85) | 152.17 (13.50) | 159.50 (14.63) |
| Lauric acid | 6189.00 (1287.32) | 6803.67 (1012.93) | 4098.84 (219.09) | 6811.67 (1344.17) |
| Levoglucosan | 589.67 (52.55) | 1122.84 (234.27) | 852.17 (168.28) | 439.67 (129.82) |
| Linoleic acid methyl ester | 465.50 (37.59) | 899.17 (488.62) | 322.34 (25.67) | 397.17 (55.00) |
| Maleic acid | 2779.50 (383.38) | 2078.00 (253.29) | 2688.00 (478.57) | 2824.34 (443.07) |
| Maleimide | 606.67 (80.56) | 713.00 (39.38) | 613.00 (64.36) | 685.00 (64.69) |
| Mannose | 579.84 (133.75) | 889.17 (147.25) | 411.17 (62.19) | 564.34 (50.98) |
| Methyl palmitoleate | 1340.34 (1024.50) | 1393.67 (1072.57) | 302.84 (36.61) | 349.50 (43.22) |
| Methylhexadecanoic acid | 5729.17 (312.34) | 5055.34 (179.22) | 4879.84 (300.63) | 5099.34 (173.73) |
| Methylmaleic acid | 172.17 (8.88) | 192.67 (18.73) | 191.84 (26.88) | 272.67 (56.57) |
| Myristic acid | 2911.50 (97.30) | 2589.67 (121.98) | 2370.50 (135.70) | 2751.17 (116.23) |
| N-acetylaspartic acid | 289.67 (111.30) | 336.84 (45.17) | 266.50 (61.21) | 188.84 (16.27) |
| N-acetyl-D-mannosamine | 207.17 (43.21) | 275.00 (38.43) | 170.17 (23.34) | 208.17 (41.06) |
| Nicotinic acid | 437.34 (52.73) | 496.50 (38.11) | 695.50 (184.04) | 497.34 (78.09) |
| Nonadecanoic acid | 785.00 (86.28) | 926.50 (194.10) | 686.00 (71.33) | 711.17 (64.34) |
| Octadecanol | 895.34 (71.32) | 803.34 (64.63) | 796.50 (119.40) | 1254.50 (283.47) |
| Oleic acid | 292.34 (25.02) | 277.17 (14.02) | 251.50 (16.37) | 268.84 (30.22) |
| Oxalic acid | 53882.67 (9087.84) | 47021.67 (6679.84) | 56965.34 (9374.91) | 67875.67 (10388.20) |
| Oxoproline | 1704.50 (442.57) | 1642.50 (206.36) | 2319.00 (578.38) | 1579.00 (184.97) |
| Palmitic acid | 22287.17 (922.90) | 23492.50 (1649.55) | 20207.84 (1434.90) | 24636.84 (756.47) |
| Pantothenic acid | 698.00 (137.61) | 1884.67 (607.77) | 864.50 (275.96) | 664.00 (256.08) |
| Parabanic acid NIST | 2406.17 (383.76) | 3885.00 (672.18) | 3081.84 (212.99) | 4398.50 (808.79) |
| Pelargonic acid | 12819.67 (6389.58) | 13583.34 (6774.19) | 6551.67 (543.07) | 6555.50 (735.22) |
| Pentadecanoic acid | 3304.00 (178.20) | 3423.17 (340.47) | 2498.67 (105.82) | 2734.00 (167.76) |
| Phenol | 1855.17 (122.41) | 1670.50 (120.14) | 1873.50 (194.61) | 1304.84 (73.32) |
| Phosphoric acid | 231.34 (41.26) | 305.00 (88.49) | 248.67 (55.69) | 239.00 (53.18) |
| Phthalic acid | 2597.17 (296.06) | 3884.67 (641.77) | 4507.00 (1166.75) | 5414.34 (1088.87) |
| P-hydroquinone | 306.67 (27.95) | 367.67 (64.87) | 314.50 (26.41) | 350.50 (37.83) |
| Phytol | 786.00 (32.94) | 668.84 (36.83) | 582.00(66.86) | 839.17 (142.00) |
| Pimaric acid NIST | 242.84 (27.29) | 292.67 (55.14) | 200.17 (32.15) | 209.67 (25.77) |
| Pimelic acid | 285.17 (27.76) | 533.34 (161.28) | 275.84 (21.41) | 328.00 (62.12) |
| Propane-1,3-diol NIST | 288.67 (37.32) | 299.00 (16.70) | 277.50 (26.35) | 224.50 (12.76) |
| Rhamnose | 1074.50 (175.54) | 3111.17 (1341.93) | 1377.34 (200.31) | 1105.50 (230.42) |
| Ribitol | 987.17 (627.87) | 667.34 (325.32) | 462.84 (141.89) | 194.50 (18.06) |
| Ribose | 162.84 (18.19) | 292.50 (81.62) | 130.17 (8.80) | 115.67 (9.36) |
| Salicylic acid | 979.84 (128.69) | 944.67 (185.98) | 1251.84 (342.73) | 1053.34 (228.81) |
| Shikimic acid | 1517.84 (234.98) | 2055.67 (313.23) | 1722.84 (249.50) | 2192.67 (340.32) |
| Stearic acid | 239381.67 (6285.32) | 247054.00 (16441.60) | 243102.17 (18111.17) | 266030.67 (11120.05) |
| Succinic acid | 1115.00 (164.74) | 1647.00 (627.90) | 2644.34 (315.75) | 2321.50 (252.41) |
| Sucrose | 158.67 (24.05) | 156.50 (22.36) | 91.34 (21.18) | 113.50(7.32) |
| Synephrine | 1499.67 (346.95) | 3166.00 (892.48) | 5852.17 (1732.75) | 4261.00 (618.74) |
| Syringic acid | 659.00 (81.00) | 797.00 (106.06) | 787.50 (153.49) | 1004.00 (149.43) |
| Tagatose | 181.00 (25.70) | 271.67 (109.70) | 120.84 (10.07) | 120.84 (10.59) |
| Terephtalic acid | 309.67 (35.81) | 279.67 (58.72) | 931.67 (255.37) | 604.84 (79.97) |
| Threitol | 878.17 (72.20) | 2288.34 (1108.09) | 856.67 (63.60) | 4442.34 (3804.26) |
| Threonine | 865.50 (45.98) | 587.17 (105.59) | 1403.50 (606.02) | 732.84 (149.40) |
| Thymidine | 328.34 (32.01) | 316.67 (53.62) | 332.34 (20.51) | 341.50 (30.79) |
| Thymine | 415.17 (56.75) | 858.50 (267.82) | 608.00 (167.40) | 230.67 (27.45) |
| Triethanolamine | 187.34 (13.52) | 179.50 (19.69) | 328.34 (71.97) | 210.50 (29.49) |
| Tyrosine | 548.17 (189.91) | 645.84 (241.73) | 700.67 (191.26) | 313.00 (23.55) |
| Urea | 14091.84 (3321.47) | 22408.17 (10443.49) | 23455.34 (4468.02) | 9384.34 (1452.26) |
| Xylitol | 371.84 (56.14) | 1226.84 (558.91) | 752.67 (192.82) | 678.17 (125.48) |
| Xylonolactone NIST | 1090.50 (158.76) | 1074.84 (205.83) | 1636.67 (430.63) | 1042.67 (128.9) |
| Xylose | 1982.34 (306.94) | 3194.84 (1117.07) | 1300.34 (366.44) | 1118.00 (225.67) |
| Xylulose NIST | 552.17 (116.80) | 1264.00 (232.78) | 840.34 (276.46) | 1517.34 (404.33) |
