# Supplementary material for: Tall fescue cultivar and fungal endophyte combinations influence plant growth and root exudate composition
Source: Front Plant Sci. 2015 Apr 9;6:183. doi: 10.3389/fpls.2015.00183 (PMC4391242; doi:10.3389/fpls.2015.00183)
Supplement: Supplementary file 4 [file Table4.DOCX]

**Table S4** Secretion level of root exudate components (identified by GC-TOF MS) released by different endophyte status and tall fescue cultivar combinations. Numbers indicate the average peak height of three replicates of each combination. Numbers in parenthesis indicate the standard error.

|  | 97TF1/E- | 97TF1/CTE+ | 97TF1/AR542E+ | 97TF1/AR584E+ | PDF/E- | PDF/CTE+ | PDF/AR542E+ | PDF/AR584E+ |
| --- | --- | --- | --- | --- | --- | --- | --- | --- |
| Amines | 9660.34 (1022.68) | 9642.34 (1145.57) | 18437.67 (1607.22) | 12003.67 (1359.20) | 7653.34 (808.23) | 12670.67 (806.56) | 11905.67 (1467.37) | 8914.67 (492.12) |
| Carboxylic acids | 110700.00 (10307.69) | 92436.00 (12437.69) | 121986.30 (8096.74) | 112749.30 (9556.97) | 100172.30 (18909.38) | 100783.00 (13243.83) | 96349.33 (16398.49) | 128555.30 (19721.24) |
| Growth factors and vitamins | 3760.00 (835.12) | 5632.67 (1518.18) | 2383.67 (128.17) | 1762.67 (368.41) | 5802.34 (1849.19) | 9420.00 (1546.99) | 7276.00 (1816.92) | 5477.34 (3807.04) |
| Lipids | 324238.70 (8802.55) | 295475.00 (25379.80) | 266249.00 (24421.35) | 338702.70 (16120.18) | 290036.30 (10876.85) | 339652.30 (16325.88) | 326437.70 (21590.75) | 317199.30 (12419.09) |
| Nucleic acids | 642.00 (62.00) | 581.34 (67.25) | 539.34 (57.46) | 729.67 (56.30) | 481.34 (62.42) | 688.34 (52.67) | 610.34 (74.11) | 483.67 (24.79) |
| Phenolics | 9851.34 (832.90) | 7420.67 (931.44) | 13395.67 (546.09) | 12424.67 (899.38) | 9609.67 (1176.27) | 17361.00 (4350.93) | 11195.00 (2666.51) | 9658.00 (633.36) |
| Polyols | 32764.33 (3152.83) | 56075.00 (4286.29) | 60402.33 (20284.14) | 31377.00 (1984.86) | 92805.00 (37555.89) | 23539.00 (5178.11) | 43182.67 (4712.20) | 42121.67 (10410.55) |
| Sugars | 10320.33 (751.73) | 26877.67 (8794.18) | 11386.67 (498.96) | 10613.33 (588.10) | 11979.67 (2081.97) | 13852.33 (1639.19) | 11232.33 (1474.56) | 9876.34 (1756.02) |
